# Supplementary material for: Non-Abelian Bloch oscillations in higher-order topological insulators
Source: Nat Commun. 2020 Nov 23;11:5942. doi: 10.1038/s41467-020-19518-x (PMC7684321; doi:10.1038/s41467-020-19518-x)
Supplement: Supplementary file 1 — Supplementary Information [file 41467_2020_19518_MOESM1_ESM.pdf]

Supplementary Information: Non-Abelian Bloch oscillations in higher-order topological insulators

M. Di Liberto,[1] N. Goldman,[1] and G. Palumbo[1]

[1] *Center for Nonlinear Phenomena and Complex Systems, Université Libre de Bruxelles, CP 231, Campus Plaine, B-1050 Brussels, Belgium*

SUPPLEMENTARY NOTE 1. SYMMETRIES AND WINDING NUMBER

The BBH model can be casted in the form

$$\hat{H}(\mathbf{k}) = \sum_{i=1}^{4} d_i(\mathbf{k})\Gamma^i = \begin{pmatrix} 0 & Q(\mathbf{k}) \\ Q(\mathbf{k})^\dagger & 0 \end{pmatrix} \tag{1}$$

$$Q(\mathbf{k}) = d_4(\mathbf{k})\mathcal{I} + i d_i(\mathbf{k})\sigma^i \,, \tag{2}$$

which explicitly shows the chiral symmetry of the model. The doubly degenerate energies are $E = \pm\epsilon_\mathbf{k}$, where $\epsilon_\mathbf{k} = \sqrt{d_1^2 + d_2^2 + d_3^2 + d_4^2}$. Moreover, notice that $Q(\mathbf{k})^\dagger = \epsilon_\mathbf{k} Q^{-1}$. The lowest two eigenstates can be written as

$$\begin{aligned} |u_1(\mathbf{k})\rangle &= \frac{1}{\sqrt{2}\epsilon_\mathbf{k}}(d_1(\mathbf{k}) - i d_2(\mathbf{k}), -d_3(\mathbf{k}) - i d_4(\mathbf{k}), 0, i\epsilon(\mathbf{k}))^T \,, \\ |u_2(\mathbf{k})\rangle &= \frac{1}{\sqrt{2}\epsilon_\mathbf{k}}(d_3(\mathbf{k}) - i d_4(\mathbf{k}), d_1(\mathbf{k}) + i d_2(\mathbf{k}), i\epsilon(\mathbf{k}), 0)^T \,, \end{aligned} \tag{3}$$

that can be compactly written as

$$v_\alpha(\mathbf{k}) = \frac{1}{\sqrt{2}} \begin{pmatrix} -Q(\mathbf{k})\xi_\alpha/\epsilon_\mathbf{k} \\ \xi_\alpha \end{pmatrix} \,, \quad \xi_1 = \begin{pmatrix} 0 \\ i \end{pmatrix}, \xi_2 = \begin{pmatrix} i \\ 0 \end{pmatrix} . \tag{4}$$

Let us consider the following non-commuting mirror symmetries $\hat{M}_x = \sigma_1 \otimes \sigma_3$ and $\hat{M}_y = \sigma_1 \otimes \sigma_1$. Without assuming a specific model we can show that a chiral symmetric Hamiltonian satisfies these mirror symmetries $\hat{M}_x \hat{H}(k_x,k_y)\hat{M}_x^{-1} = \hat{H}(-k_x,k_y)$ and $\hat{M}_y \hat{H}(k_x,k_y)\hat{M}_y^{-1} = \hat{H}(k_x,-k_y)$ if and only if

$$\begin{aligned} d_1(k_x,k_y) &\overset{\hat{M}_x}{=} +d_1(-k_x,k_y) \,, \\ d_2(k_x,k_y) &\overset{\hat{M}_x}{=} +d_2(-k_x,k_y) \,, \\ d_3(k_x,k_y) &\overset{\hat{M}_x}{=} -d_3(-k_x,k_y) \,, \\ d_4(k_x,k_y) &\overset{\hat{M}_x}{=} +d_4(-k_x,k_y) \,, \end{aligned} \tag{5}$$

and

$$\begin{aligned} d_1(k_x,k_y) &\overset{\hat{M}_y}{=} -d_1(k_x,-k_y) \,, \\ d_2(k_x,k_y) &\overset{\hat{M}_y}{=} +d_2(k_x,-k_y) \,, \\ d_3(k_x,k_y) &\overset{\hat{M}_y}{=} +d_3(k_x,-k_y) \,, \\ d_4(k_x,k_y) &\overset{\hat{M}_y}{=} +d_4(k_x,-k_y) \,. \end{aligned} \tag{6}$$

We also consider the $\hat{C}_4$ symmetry, namely $\hat{C}_4 \hat{H}(k_x,k_y)\hat{C}_4^{-1} = \hat{H}(k_y,-k_x)$, represented by

$$\hat{C}_4 = \begin{pmatrix} 0 & \mathcal{I} \\ -i\sigma_2 & 0 \end{pmatrix} . \tag{7}$$

This symmetry translates into

$$\begin{aligned} d_1(k_x,k_y) &\overset{\hat{C}_4}{=} +d_3(k_y,-k_x) \,, \\ d_2(k_x,k_y) &\overset{\hat{C}_4}{=} +d_4(k_y,-k_x) \,, \\ d_3(k_x,k_y) &\overset{\hat{C}_4}{=} -d_1(k_y,-k_x) \,, \\ d_4(k_x,k_y) &\overset{\hat{C}_4}{=} +d_2(k_y,-k_x) \,. \end{aligned} \tag{8}$$

We will now demonstrate that, along the closed path $\mathcal{C}$, the previous symmetries quantize the following quantity

$$\begin{aligned} w_c &= \frac{i}{2\pi}\int_\mathcal{C} \mathrm{d}\mathbf{k}\, \mathrm{Tr}\left[Q(\mathbf{k})^{-1}\sigma_3\, \partial_\mathbf{k} Q(\mathbf{k})\right] \\ &= -\frac{1}{\pi}\int_\mathcal{C} \mathrm{d}\mathbf{k}\, \frac{1}{\epsilon_\mathbf{k}}\left[d_1(\mathbf{k})\partial_\mathbf{k} d_2(\mathbf{k}) - d_2(\mathbf{k})\partial_\mathbf{k} d_1(\mathbf{k})\right. \\ &\quad \left. + d_3(\mathbf{k})\partial_\mathbf{k} d_4(\mathbf{k}) - d_4(\mathbf{k})\partial_\mathbf{k} d_3(\mathbf{k})\right] \,, \end{aligned} \tag{9}$$

and that such a quantity is a winding number. Let us now focus on the path $\mathcal{C}$ and use the following hypothesis

$$d_1 = d_1(k_y)\,, d_2 = d_2(k_y)\,, d_3 = d_3(k_x)\,, d_4 = d_4(k_x)\,, \tag{10}$$

namely that the d_i vectors are functions of only one momentum component, which is satisfied by the BBH model. Then the integrand of w_c can be written as

$$w_c = -\frac{1}{\pi}\int_0^{2\pi} \frac{\mathrm{d}k}{\epsilon_k^2} w_c^{(x)} - \frac{1}{\pi}\int_0^{2\pi} \frac{\mathrm{d}k}{\epsilon_k^2} w_c^{(y)} \,. \tag{11}$$

We can calculate the two terms separately

$$\begin{aligned} w^{(x)} &= d_1(\mathbf{k})\partial_{k_x} d_2(\mathbf{k}) - d_2(\mathbf{k})\partial_{k_x} d_1(\mathbf{k}) + d_3(\mathbf{k})\partial_{k_x} d_4(\mathbf{k}) - d_4(\mathbf{k})\partial_{k_x} d_3(\mathbf{k}) \\ &\overset{(10)}{=} d_3(k_x,k_y)\partial_{k_x} d_4(k_x,k_y) - d_4(k_x,k_y)\partial_{k_x} d_3(k_x,k_y) \\ &\overset{(8)}{=} -d_1(k_y,-k_x)\partial_{k_x} d_2(k_y,-k_x) + d_2(k_y,-k_x)\partial_{k_x} d_1(k_y,-k_x) \\ &\overset{(6)}{=} d_1(k_y,k_x)\partial_{k_x} d_2(k_y,k_x) - d_2(k_y,k_x)\partial_{k_x} d_1(k_y,k_x) \\ &\overset{(10)}{=} d_1(k)\partial_k d_2(k) - d_2(k)\partial_k d_1(k)\,. \end{aligned} \tag{12}$$

Analogously, for the other term

$$\begin{aligned} w^{(y)} &= d_1(\mathbf{k})\partial_{k_y} d_2(\mathbf{k}) - d_2(\mathbf{k})\partial_{k_y} d_1(\mathbf{k}) + d_3(\mathbf{k})\partial_{k_y} d_4(\mathbf{k}) - d_4(\mathbf{k})\partial_{k_y} d_3(\mathbf{k}) \\ &\overset{(10)}{=} d_1(k_x,k_y)\partial_{k_y} d_2(k_x,k_y) - d_2(k_x,k_y)\partial_{k_y} d_1(k_x,k_y) \\ &= d_1(k)\partial_k d_2(k) - d_2(k)\partial_k d_1(k)\,. \end{aligned} \tag{13}$$

We then find after noticing that $d_1(k) = d_3(k)$ and $d_2(k) = d_4(k)$ (which we justify below based on the combination of $\hat{C}_4$ and $\hat{M}_y$ symmetries)

$$w_c = -\frac{1}{\pi}\int_0^{2\pi} \mathrm{d}k\, \frac{d_1(k)\partial_k d_2(k) - d_2(k)\partial_k d_1(k)}{|d_1(k)|^2 + |d_2(k)|^2}\,. \tag{14}$$

For the BBH model we obtain

$$w_c = \mathrm{sign}(J_1^2 - J_2^2). \tag{15}$$

Let us now consider the combination of $\hat{C}_4$ and $\hat{M}_y$, namely $\hat{M}_y C_4 \hat{H}(k_x,k_y)\hat{C}_4^{-1}\hat{M}_y^{-1} = \hat{M}_y \hat{H}(k_y,-k_x)\hat{M}_y^{-1} = \hat{H}(k_y,k_x)$, which is nothing else than a mirror symmetry with respect to the diagonal axis. This condition constrains the vectors d_i as follows. Let us consider in particular the set of points $k_x = k_y$. By explicitly calculating the $\hat{M}_y\hat{C}_4$ mirror symmetry condition at $k_x = k_y$ for a Dirac Hamiltonian respecting (10), we immediately find that $d_1(k) = d_3(k)$ and $d_2(k) = d_4(k)$.

The last task is to connect the winding number with the Wilson loop operator. Let us now consider the Berry connection

$$\begin{aligned} A_x^{12}(\mathcal{C}) &= i\langle u_1(\mathbf{k})|\partial_{k_x} u_2(\mathbf{k})\rangle_{\mathcal{C}} \\ &= \frac{1}{2\epsilon_k^2}\big[(d_3 - id_4)\partial_{k_x}(d_2 - id_1) \\ &\quad +(d_1 + id_2)\partial_{k_x}(id_3 + d_4)\big]_{\mathcal{C}} \\ &= \frac{1}{2\epsilon_k^2}\left[(d_1 + id_2)\partial_k(id_1 + d_2)\right]_{k_x=k_y=k} \\ &= \frac{1}{2\epsilon_k^2}(d_1\partial_k d_2 - d_2\partial_k d_1) + \frac{i}{2\epsilon_k^2}(d_1\partial_k d_1 + d_2\partial_k d_2)\,. \end{aligned} \tag{16}$$

The y component reads

$$\begin{aligned} A_y^{12}(\mathcal{C}) &= i\langle u_1(\mathbf{k})|\partial_{k_y} u_2(\mathbf{k})\rangle_{\mathcal{C}} \\ &= \frac{1}{2\epsilon_k^2}\big[(d_3 - id_4)\partial_{k_y}(d_2 - id_1) \\ &\quad +(d_1 + id_2)\partial_{k_y}(id_3 + d_4)\big]_{\mathcal{C}} \\ &= \frac{1}{2\epsilon_k^2}(d_1 - id_2)\partial_k(d_2 - id_1) \\ &= \frac{1}{2\epsilon_k^2}(d_1\partial_k d_2 - d_2\partial_k d_1) - \frac{i}{2\epsilon_k^2}(d_1\partial_k d_1 + d_2\partial_k d_2)\,. \end{aligned} \tag{17}$$

We therefore find that

$$\int_{\mathcal{C}} (\mathrm{d}k_x A_x^{12} + \mathrm{d}k_y A_y^{12}) = \frac{1}{2}\int_0^{2\pi} \mathrm{d}k \frac{d_1\partial_k d_2 - d_2\partial_k d_1}{|d_1|^2 + |d_2|^2} = -\frac{\pi}{2} w_c\,. \tag{18}$$

The other component of the Berry connection reads

$$\begin{aligned} A_x^{21}(\mathcal{C}) &= i\langle u_2(\mathbf{k})|\partial_{k_x} u_1(\mathbf{k})\rangle_{\mathcal{C}} \\ &= \frac{1}{2\epsilon_k^2}\big[(d_3 + id_4)\partial_{k_x}(d_2 + id_1) \\ &\quad +(d_1 - id_2)\partial_{k_x}(-id_3 + d_4)\big]_{\mathcal{C}} \\ &= \frac{1}{2\epsilon_k^2}(d_1 - id_2)\partial_{k_x}(-id_3 + d_4) \\ &= \frac{1}{2\epsilon_k^2}(d_1 - id_2)\partial_k(-id_1 + d_2) \\ &= \frac{1}{2\epsilon_k^2}(d_1\partial_k d_2 - d_2\partial_k d_1) - \frac{i}{2\epsilon_k^2}(d_1\partial_k d_1 + d_2\partial_k d_2)\,, \end{aligned} \tag{19}$$

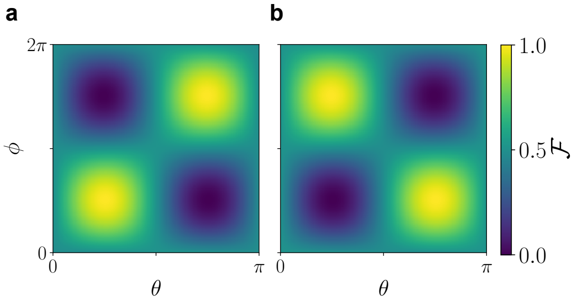

Supplementary Figure 1. Fidelity of **(a)** the ground state, $\mathcal{F}^1$, and **(b)** of the excited state, $\mathcal{F}^2$, as a function of the BBH eigenstates degenerate manifold for flux $\varphi = \pi - 0.1$ and $J_2 = 0.5J_1$.

whereas

$$\begin{aligned} A_y^{21}(\mathcal{C}) &= i\langle u_2(\mathbf{k})|\partial_{k_y} u_1(\mathbf{k})\rangle_{\mathcal{C}} \\ &= \frac{1}{2\epsilon_k^2}\big[(d_3 + id_4)\partial_{k_y}(d_2 + id_1) \\ &\quad + (d_1 - id_2)\partial_{k_y}(-id_3 + d_4)\big]_{\mathcal{C}} \\ &= \frac{1}{2\epsilon_k^2}(d_3 + id_4)\partial_{k_y}(d_2 + id_1) \\ &= \frac{1}{2\epsilon_k^2}(d_1 + id_2)\partial_{k_y}(d_2 + id_1) \\ &= \frac{1}{2\epsilon_k^2}(d_1\partial_k d_2 - d_2\partial_k d_1) + \frac{i}{2\epsilon_k^2}(d_1\partial_k d_1 + d_2\partial_k d_2)\,, \end{aligned} \tag{20}$$

and we finally conclude that

$$\int_{\mathcal{C}} (\mathrm{d}k_x A_x^{21} + \mathrm{d}k_y A_y^{21}) = -\frac{\pi}{2} w_c\,. \tag{21}$$

Moreover, notice that $A_i^{12}(\mathbf{k}) = [A_i^{21}(\mathbf{k})]^*$ as required by SU(2).

Let us now have a look at the diagonal components of the Berry connection

$$\begin{aligned} A_x^{11}(\mathcal{C}) &= \frac{1}{2\epsilon_k^2}\left[d_1\partial_{k_x}d_2 - d_2\partial_{k_x}d_1 - d_3\partial_{k_x}d_4 + d_4\partial_{k_x}d_3\right]_{\mathcal{C}} \\ &= \frac{1}{2\epsilon_k^2}(-d_3\partial_k d_4 + d_4\partial_k d_3) \\ &= \frac{1}{2\epsilon_k^2}(-d_1\partial_k d_2 + d_2\partial_k d_1)\,, \end{aligned} \tag{22}$$

whereas

$$\begin{aligned} A_y^{11}(\mathcal{C}) &= \frac{1}{2\epsilon_k^2}\left[d_1\partial_{k_y}d_2 - d_2\partial_{k_y}d_1 - d_3\partial_{k_x}d_4 + d_4\partial_{k_x}d_3\right]_{\mathcal{C}} \\ &= \frac{1}{2\epsilon_k^2}(d_1\partial_k d_2 - d_2\partial_k d_1) \end{aligned} \tag{23}$$

thus concluding that $A_x^{11}(\mathcal{C}) + A_y^{11}(\mathcal{C}) = 0$ which shows that the Wilson loop on the path $\mathcal{C}$ is only off-diagonal, and in particulare that

$$\int_{\mathcal{C}} \mathrm{d}\mathbf{k}\cdot\mathbf{A}(\mathbf{k}) = \pm\frac{\pi}{2}\sigma_1\,. \tag{24}$$

By using crystal symmetries and the combination $\hat{C}_4\hat{M}_x$, similar relations can be obtained for the $\bar{\mathcal{C}}$ path, where we find that

$$\int_{\bar{\mathcal{C}}} \mathrm{d}\mathbf{k}\cdot\mathbf{A}(\mathbf{k}) = \pm\frac{\pi}{2}\sigma_3\,. \tag{25}$$

SUPPLEMENTARY NOTE 2. DEGENERACY BREAKING

Here, we consider a state preparation protocol based on the breaking of time-reversal symmetry in the BBH model. Let us consider the vertical hopping coefficients responsible for the π flux to have a generic complex dependence $e^{i\varphi}$, corresponding to plaquettes with staggered flux $\pm\varphi$. The eigenstates at the Γ point read

$$\begin{aligned} |u_\Gamma^1(\varphi)\rangle &= \frac{1}{2}\left(|\sin(\varphi/4)|(1 + i\cot(\varphi/4)), \frac{4|\sin(\varphi/4)|\cos(\varphi/2)}{2\cos(\varphi/2) - \cos\varphi + i\sin\varphi - 1}, -\cos(\varphi/2) + i\sin(\varphi/2), 1\right)^T\,, \\ |u_\Gamma^2(\varphi)\rangle &= \frac{1}{2}\left(|\cos(\varphi/4)|(1 - i\tan(\varphi/4)), \frac{4|\cos(\varphi/4)|\cos(\varphi/2)}{2\cos(\varphi/2) + \cos\varphi - i\sin\varphi + 1}, \cos(\varphi/2) - i\sin(\varphi/2), 1\right)^T\,, \end{aligned} \tag{26}$$

with energies $E_1 = -(J_1 + J_2)|\sin(\varphi/4)|$ and $E_2 = -(J_1 + J_2)|\cos(\varphi/4)|$. Let us now consider a generic combination of the π flux eigenstates as considered in the main text, namely $|u_\Gamma(\theta,\phi)\rangle = \cos(\theta)|u_\Gamma^1\rangle + \sin(\theta)e^{i\phi}|u_\Gamma^2\rangle$. For $\varphi = \pi - 0.1$, the lowest energy state is $|u_\Gamma^1(\varphi)\rangle$ and we can then calculate the fidelity $\mathcal{F}^\alpha = |\langle u_\Gamma(\theta,\phi)|u_\Gamma^\alpha(\varphi)\rangle|^2$, which is shown in Supplementary Figure 1. We therefore find that the ground state is a distribution of the degenerate BBH eigenstates peaked at $\theta = \pi/4$, $3\pi/4$ and $\phi = \pi/2$, $3\pi/2$. An analogous reasoning can be repeated for the excited state $|u_\Gamma^2(\varphi)\rangle$.

A protocol for state preparation would then require to slightly break time-reversal symmetry in order to prepare a BEC occupying the ground state $|u_\Gamma^1(\varphi)\rangle$. Then, one can treat the states $|u_\Gamma^{1,2}(\varphi)\rangle$ as a two-level system and apply a coherent external coupling with frequency

$\omega = \Delta E = E_2 - E_1$ to make a superposition of $|u_\Gamma^1(\varphi)\rangle$ and $|u_\Gamma^2(\varphi)\rangle$ with relative imbalance (parametrized by θ) and phase (parametrized by ϕ) as the initial states discussed in the main text. In order to reproduce the BOs results discussed in this work, the applied force must then satisfy $|F| \gg \Delta E$ such that the two bands are effectively degenerate on the time-scale of the BO.
